# Cattle Manure Trade Network Analysis and the Relevant Spatial Pathways in an Endemic Area of Foot and Mouth Disease in Northern Thailand

**DOI:** 10.3390/vetsci7030138

**Published:** 2020-09-19

**Authors:** Chalutwan Sansamur, Anuwat Wiratsudakul, Arisara Charoenpanyanet, Veerasak Punyapornwithaya

**Affiliations:** 1 Department of Veterinary Bioscience and Veterinary Public Health, Faculty of Veterinary Medicine, Chiang Mai University, Chiang Mai 50100, Thailand; chalutwan_s@cmu.ac.th; 2 Department of Clinical Sciences and Public Health and The Monitoring and Surveillance Center for Zoonotic Diseases in Wildlife and Exotic Animals, Faculty of Veterinary Science, Mahidol University, Phutthamonthon, Nakhon Pathom 73170, Thailand; anuwat.wir@mahidol.edu; 3 Department of Geography, Faculty of Social Sciences, Chiang Mai University, Chiang Mai 50200, Thailand; arisara.cmu@gmail.com; 4 Veterinary Public Health and Food Safety Centre for Asia Pacific (VPHCAP), Faculty of Veterinary Medicine, Chiang Mai University, Chiang Mai 50100, Thailand

**Keywords:** cattle manure, manure trade movement, network analysis, foot and mouth disease, cattle farms, Northern Thailand

## Abstract

Animal movement is one of the most important risk factors for outbreaks of foot and mouth disease (FMD) in cattle. Likewise, FMD can spread to cattle farms via vehicles contaminated with the FMD virus. In Northern Thailand, the movement of manure transport vehicles and the circulation of manure bags among cattle farms are considered as potential risk factors for FMD outbreaks among cattle farms. This study aimed to determine the characteristics and movement patterns of manure tradesman using social network analysis. A structured questionnaire was used to identify sequences of farms routinely visited by each tradesman. A total of 611 participants were interviewed, including 154 beef farmers, 407 dairy farmers, 36 tradesmen, and 14 final purchasers. A static weighted directed one-mode network was constructed, and the network metrics were measured. For the manure tradesman–cattle farmer network, the tradesman possessed the highest value of in- and out-degree centralities (71 and 4), betweenness centralities (114.5), and k-core values (2). These results indicated that the tradesman had a high frequency of farm visits and had a remarkable influence on other persons (nodes) in the network. The movement of vehicles ranged from within local districts, among districts, or even across provinces. Unclean manure plastic bags were circulated among cattle farms. Therefore, both vehicles and the bags may act as a disease fomite. Interestingly, no recording system was implemented for the movement of manure transport vehicles. This study suggested that the relevant authority and stakeholders should be aware of the risk of FMD spreading within this manure trading network. The findings from this study can be used as supporting data that can be used for enhancing FMD control measures, especially for FMD endemic areas.

## 1. Introduction

Foot and mouth disease (FMD), a highly contagious infectious disease, has a major economic impact on livestock trade [1]. At the global level, the costs of production losses and vaccine costs are assumed to be in the range of US$6.5–21 billion, which directly affects overall food security in endemic countries [2]. At the local community level, animal morbidity results in the largest amount of financial losses [3]. Notably, FMD affects mainland Southeast Asia, namely Lao People’s Democratic Republic, Cambodia, Myanmar, Vietnam, Malaysia, and Thailand [4]. There were 4930 FMD outbreaks in the period 2007 to 2016 [5]. A variety of livestock production has been commonly practiced in Northern Thailand including dairy and beef cattle. In this area, FMD outbreaks gradually increased. The accumulative number of outbreaks was 140 between 2008 and 2015 [6]. Nonetheless, the situation was worsened in 2016. In this particular year, 73 outbreaks were reported [7].

FMD affects cloven-hoofed animals, such as cattle, sheep, goats, pigs, and wild boar. The virus causes noticeable vesicles on feet, mammary glands, and around the oral cavity of infected animals [8]. It has been established well that the FMD virus (FMDV) is transmitted through direct or indirect contact between infected and susceptible animals, their secretions, and contaminated animal products [9]. As evidenced by various previous studies, the movement of animals was significantly associated with FMD outbreaks [10,11,12]. The movement of live cattle may result in the introduction of the FMDV onto a farm. For example, contamination may occur via the purchase of new cattle from infected farms and premises [13] and the movement of live cattle during an outbreak [14]. Cattle movement is not only a risk in and of itself, but contamination can also occur as a result of indirect or mechanical spread from farm to farm via contaminated vehicles as well as their secretions, urine, and feces (cattle manure) [15]. As for the risk of indirect transmission, the FMDV can contaminate the environment and be transmitted through fomites on vehicles, equipment, feed, persons [16,17], and the unrestricted movements of people or vehicles such as live cattle tradesman [18].

In Northern Thailand, one of the most common agricultural activities is the trading of cattle manure. Manure tradesmen visit cattle farms to buy cattle manure filled in used concentrate cattle feed bags and then transport manure to final purchasers who use manure as a fertilizer for crops. The spread of FMDV may occur via contaminated vehicles or manure bags because FMDV can survive on such fomites for many days [15]. Regarding FMD control measures, authorities from the Department of Livestock Development announced that the movement of manure transport vehicles should be restricted during FMD outbreaks as this activity may involve the spreading of disease [19]. However, there is a knowledge gap, as no studies have examined the movement inside manure trade networks, making it difficult to track such movement.

Social network analysis (SNA) has been applied as an important methodological approach of veterinary epidemiology to document contact patterns among interacting individuals and to characterize animal movement as it relates to the spread of disease [20,21,22]. Studies using SNA have shown that farmers, cattle tradesmen, and animal markets were key players in the spread of FMD among farm animals, and particular attention should be paid to these important nodes when attempting to reduce future FMD outbreaks [23,24,25]. Similar to cattle movement, the trading activity of manure tradesman, cattle farmers, and final purchasers can be considered as a network.

It is beneficial to determine and explore the network of manure trading as the transmission of FMDV can occur through network activities. In addition, understanding the route and frequency of movements is important for livestock authorities and stakeholders to develop effective control strategies, particularly for the endemic FMD area. Therefore, the aim of this study was to determine the characteristics of the manure trade network and movement pathways of manure transport vehicles.

## 2. Materials and Methods

### 2.1. Study Area

Mae On, San Kamphaeng, and San Sai districts in Chiang Mai province were chosen for this study based on the agricultural importance of these areas in terms of dairy production and FMD outbreak notifications. Beef cattle are also found to be ubiquitously present in these districts. In addition, plenty of livestock products and byproducts, such as milk and manure, are produced by the smallholders that are located in these areas.

### 2.2. Data Sources

A list of beef (*n* = 154) and dairy farms (*n* = 407) was obtained from Chiang Mai Provincial Livestock Office and dairy cooperatives. Data consisted of farm owner name, farm identification number, and farm location.

### 2.3. Questionnaire Survey

Two specific questionnaires (Supplementary Materials) were used to collect data from cattle farm owners and manure tradesmen. The farm owners identified from either livestock authority or the dairy cooperative lists were interviewed with questionnaires via face-to-face communication. The snowball sampling method [26] was used in order to find manure tradesmen. The sampling works like a chain referral as we identified an initial tradesman and then we asked that person to nominate another tradesman and continued in the same way. The survey covered activities that took place from February through June 2017. For cattle farm owners, the survey questions included general demographic data of the respondents, the frequency of manure trading activities, and locations where the manure was sold. Manure tradesmen were interviewed with regard to the frequency of manure trading, relevant interpersonal connections, and intended destinations of the manure being delivered. All participants agreed to voluntarily take part in the study and offered their informed consent to participate prior to being interviewed.

### 2.4. Statistical Analysis

A static weighted directed one-mode network was constructed. A node referred to a dairy farmer, a beef farmer, or a tradesman in the manure network, while a tie referred to manure trading activity. The direction of each tie was set according to the corresponding direction of its trading relationship and the width was defined in relation to its weight. The weight of the ties was defined by the frequency of the manure trading activity. The manure trade network was analyzed at the node level, in subgroups, and by the entire network. The main network parameters are described in Table 1. Network analyses were performed using R version 3.6.2 (R Core Team, Vienna, Austria) [27] with the packages “igraph” version 0.7.1. [28], “tnet” version 3.0.11 [29], and “cluster” version 2.1.0 [30]. Euclidean distances between nodes were measured with the package “magrittr” version 1.5 [31]. Geographical and attributed data obtained from the Chiang Mai Provincial Livestock Office were used to construct a disease map using QGIS version 2.18.28 (Open Source Geospatial Foundation Project, Zurich, Switzerland) [32].

## 3. Results

### 3.1. Description of Respondents

In total, 611 individuals from the three studied districts responded to our questionnaire (Mae On = 380, San Kamphaeng = 155, and San Sai = 76). Around two-thirds of the respondents were dairy farmers. Most respondents were male (84.6%), while approximately half of the participants were older than 40 years old. Additionally, around 75% were educated at the primary school level. The roles and number of participants are shown in Table 2. Sixty percent of manure tradesmen were based in Mae On district. Most dairy farms and beef farms were located in Mae On, San Kamphaeng, and San Sai district, respectively. The average number of beef cattle was in the range of 7–10 animals per farm, whereas the average number of dairy cattle per farm was around 32–36 animals.

### 3.2. Manure Trading, Transportation and Bio-Security

**Figure 1:**
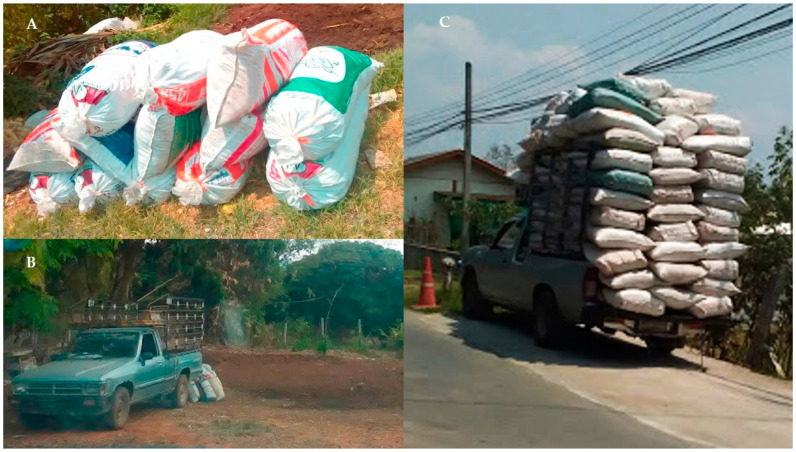
Manure bags that were circulated and reused (**A**), the entrance of manure tradesmen’s vehicle in cattle farm (**B**), and manure transportation (**C**) (photos taken by C. Sansamur).

Manure tradesmen traveled to beef and dairy farms to collect manure packed in used concentrate cattle feed bags all year without any specified or pre-appointed routes. The average frequency of visits to cattle farms by a manure tradesman was 0.48 ± 0.22 times per month. The average frequency of visits to beef and dairy farms by a manure tradesman was 0.32 ± 0.06 and 0.53 ± 0.14 times per month, respectively. None of the tradesmen cleaned the vehicle with a disinfectant agent. Approximately 50–100 manure bags (Figure 1) were repeatedly used and circulated among cattle farms each day. The manure bags had repeatedly been used and circulated among cattle farms without cleaning.

### 3.3. Movement Pathway of Manure Transportation

**Figure 2:**
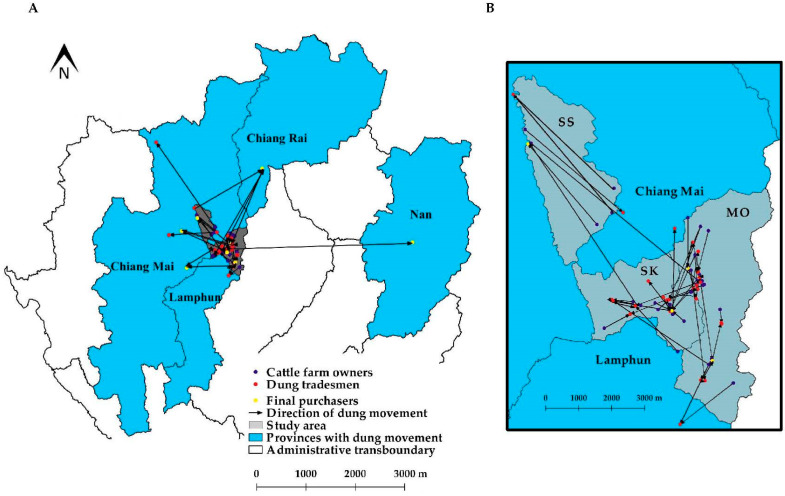
Map of Northern Thailand depicting spatial distribution of manure movements in the network (**A**) and the magnified map focusing manure movements on the study area including Mae On (MO), San Kamphaeng (SK), and San Sai (SS) (**B**).

This study found a variety of movement pathways since tradesmen have no regular schedule to visit cattle farms. The movement of manure may range from within local districts, among districts, or even across provinces (Figure 2). The shortest distance of the manure movement was less than 1 km, whereas the longest distance was over 100 km (Table 3). In contrast to livestock animal transport, which requires permission from the livestock officer, the transport of manure is unrestricted and no transportation data are kept.

### 3.4. Characterization of Manure Trade Network

**Figure 3:**
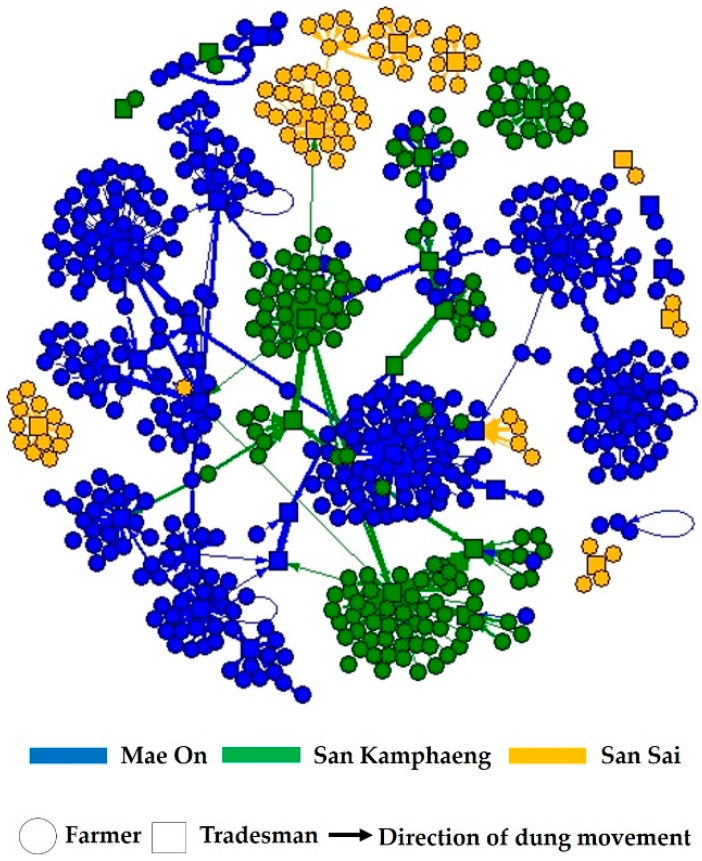
Static weighted directed one-mode network of manure movement in Mae On (blue), San Kamphaeng (green), and San Sai (yellow) district. Arrowheads indicate the direction of manure movements. The thickness of the ties was directly related to the weight of the ties.

A static weighted directed one-mode network was employed to illustrate the manure tradesmen–cattle farmers network of manure trading within the three districts (Figure 3). This network of manure trade is made up of 611 nodes and 629 weighted ties. The numbers of nodes for the network made up of Mae On, San Kamphaeng, and San Sai districts were 380, 155, and 76, respectively. The average number of sub-networks of tradesmen had 24 memberships. The largest sub-network of tradesmen had 72 memberships, including 67 dairy farm owners, 3 beef farm owners, and two tradesmen. In addition, some farmers (*n* = 38) sold manure to multiple tradesmen. Moreover, the final purchasers (*n* = 3) bought manure from multiple tradesmen.

The average in-degree centrality value for all nodes was 1.03 (SD = 5.61), which was similar to the out-degree centrality (1.03, SD = 0.35). The average betweenness and closeness centralities of all nodes were 0.67 (SD = 6.83) and 2.69 × 10^−6^ (SD = 4.72 × 10^−9^), respectively. For node level, the node with the highest in- and out-degree, weighted in-degree, and betweenness centralities was a tradesman type (Table 4). In the part of the sub-network level, the average k-core value was 1.1 (range = 1–2). The social network parameters of the different groups of nodes in the manure trade network of the three districts are detailed in Table 4.

The majority of the dyads in the network were classified into the null group (185,729 pairs). The numbers of mutual and asymmetric dyads were relatively low at 3 and 623 pairs, respectively. Likewise, most triads were counted as empty graphs (37,460,585 triads), followed by triads with a single directed tie (359,556 triads) and triads with the in-star type (9511 triads). No completely connected triads were observed in the network. The dyad and triad census and frequency distribution values are shown in Table 5 and Table 6, respectively.

The network level, the density of the whole network was 0.0016, and the clustering coefficient of this network was 0.027. There were 15 weakly connected components (WCC) in this network. The major WCC containing 528 nodes (86.4%) was found at the center of the network. The 14 remaining WCC were relatively small and disconnected from the major WCC.

## 4. Discussion

Previous studies conducted in Thailand, Laos, and Cambodia indicated that cattle tradesmen and their movements were key factors in the spread of infectious diseases within the cattle trade network [39,40,41]. In Thailand, especially in the northern region, manure tradesmen conduct some activities similar to cattle tradesmen. For example, they routinely visit multiple cattle farms on a daily basis and some travel further to other provinces. Therefore, there is an opportunity for the FMDV to spread among cattle farms via the contaminated manure bags and/or vehicles of tradesmen. In this study, we determined the network of manure tradesman–cattle farmers and explored the movement pathways of manure transport.

With a high value of in- and out-degree, weighted in-degree, and betweenness centralities, tradesman was the most important node in the manure tradesmen–cattle farmers network (Table 4). These tradesmen connected with many farm owners for manure trading and they also linked with different final purchasers, as illustrated in Figure 2. This finding was similar to what was observed in previous studies [27,42]. This suggested that the nodes found in the cattle movement network influenced how FMDV spread by linking members of the network during an outbreak. Our study also implied that most cattle farm owners sold manure to a specific tradesman. In terms of frequency, dairy farm owners had a higher frequency of manure trading activities than beef farm owners. This finding is explainable by the fact that beef cattle are generally raised in a free-ranging fashion during the daytime. In contrast, dairy cattle are reared within the farms, resulting in a higher amount of accumulated manure.

Within the manure trade network, the subgroup connection has a large weak component that contains 86.4% of nodes. This suggests that some members can be reached by others, but most members were not. The weak component has the advantage that control measures are potentially more efficient than networks with strong components [43]. Contrarily, the large strong component network has the potential to increase the size of an epidemic; thus, the control measures have to be enhanced as described in previous studies [11,42,44]. Similar to a previous study in Northern Thailand [45], we found that most of the dyads census were null and most of the triads were empty (Table 5 and Table 6). These results indicate that the network possesses unconnected property. The null dyad refers to no manure trading among cattle farm owners. In other words, the movement of the vehicle from one farm to another for manure transportation is not generally observed. Additionally, both network density and clustering coefficient were relatively low, suggesting that the networks were likely to be characterized by a random pattern.

Animal movement restriction is a key FMD control strategy in Thailand [46]. At the provincial level, the movements of live cattle across provinces are routinely checked based on official movement documents [47]. However, the vehicle movement of the manure tradesman is not required to be recorded. Although livestock authorities recommend that the movement of dung transport vehicles should be restricted [19], little is known about the frequency and pathway of trade. Therefore, this information gap could affect the development of effective control measures, as the movement of illegal manure transport vehicles could occur.

This study identified that there is no common movement pathway for manure tradesmen as the traveling schedule depends on an everyday phone call by the cattle owners and the final purchaser. Most traveling occurred within the district and across districts (Table 3). Interestingly, the tradesmen used the same pathways used by dairy farmers to transport farm bulk tank milk to milk collecting centers. Furthermore, some tradesmen traveled to other districts or neighboring provinces of Chiang Mai (Figure 2). Several tradesmen traveled back and forth between large dairy farming zones in Chiang Mai and Lamphun. Thus, manure tradesmen may act as a fomite for FMD transmission across a province boundary. As a result, it can be concluded that, with a wide range of tradesmen’s movement pathways and high connectivity between tradesmen and cattle farm owners, manure tradesmen may accommodate the spread of FMD within the network.

Based on the findings from this study, management practices for the prevention of FMDV spread from manure trade activities should be performed in addition to the current FMD control practices. For example, farmers should prepare a disinfecting area for the vehicles of manure tradesman at the farm’s entrance. A logbook should be kept to record the contact information of manure tradesmen and trading activities such as the date and time of the visit and the number of manure bags sold. In addition, an educational campaign on the risk of FMD and biosecurity should be prepared for the manure tradesmen. Importantly, manure bags should not be circulated among cattle farms. In other words, the bags should be discarded after their first use as they can be contaminated with the FMDV and become another source of indirect FMDV transmission.

Approaches used in this study may be useful for researchers in some agricultural producing countries that have manure trading, such as countries in Africa [48] and Asia [49]. The SNA technique can be extended to other forms of movement, such as movements of animals, feed vehicles [17], and cattle traders [43] corresponding to farm visits. This technique may also be applied to other diseases or health conditions.

To the best of our knowledge, this study is the first report to investigate the characteristics of the cattle manure network and movement pathways of manure tradesmen in Northern Thailand. However, this study has some limitations. Firstly, the relationship between the vehicle movements of manure tradesmen and the occurrence of FMD outbreaks could not be determined because data on the date and time of the manure tradesmen visit to the farm were not recorded. We suggest that the association between manure trading and FMD outbreak should be elaborately investigated for future studies. Secondly, the determination of the static network was based on a cross-sectional design study; therefore, a temporal trend could not be determined. For follow-up studies, the dynamic network accounting for the temporal variations in the trade volume at different times of the year should be determined.

## 5. Conclusions

In the present study, the manure tradesmen–cattle famers network was identified and the movement pathways due to transportation activities were illustrated. We intended to raise awareness among stakeholders and relevant authorities concerning the likelihood of FMD dissemination through this manure trading network. The findings of this study might be useful for livestock authorities, cattle farmers, and manure tradesmen to develop an enhanced control strategy for FMD. The approach used in this study may be applied to other types of movements or activities that may contribute to the spread of FMDV if data are presented as a network formation.

## Figures and Tables

**Table 1:** Description of the network parameters used in this study.

Parameter	Description
**Node Level**	
Degree centrality	Degree centrality was subdivided into in-degree, which refers to the number of contacts that a node receives, and out-degree centrality, which refers to the number of contacts that originated from a node [33]. In this study, both degree centralities were measured. In-degree centrality represents the number of incoming ties of the cattle farm owners and manure tradesman. Out-degree centrality measures the number of outgoing ties of manure trade activities. Weighted in- and out-degree were defined as the frequency of manure movement, incoming and outgoing ties, respectively.
Betweenness centrality	The centrality extent to which a node lies between other nodes in the network. It measures the frequency with which a node is in the shortest path between each node pair, which connect indirectly through their direct ties [33,34]. In this study, the node with the highest value indicates a measurement of the node’s influence.
Closeness centrality	It is the inverse of farness or the average distances between each and all other nodes in the network [33,34]. This centrality is the average of the shortest path length from one node to every other node in the manure trade network.
**Subgroup level**	
*K-core*	*K*-core is referred to the minimum number of connections of each node which is connected to at least *k* nodes in the subgroup [35]. In this study, *k-core* refers to the minimum number of cattle farm owners and tradesmen that all the members of the subgroup belonging to the focal node have. The *k-core* values were calculated using algorithms explained in a previous study [36].
Dyads and triads	A dyad is a unit of two nodes which are probably tied with each other. In a directed network, there are three isomorphism classes of dyads including mutual (A<->B), asymmetric (A->B), and null. A triad is a subgroup of three nodes (A, B, C) and ties between these nodes. Triads in a directed graph may belong to one of 16 isomorphism classes by the number of ties presented [37].
Component	Maximally connected nodes that can reach one another by at least one tie directly [34].
**Network level**	
Density	A proportion of the contacts between pairs of nodes [37]. This is possibly represented in the manure trade network compared with those that were actually observed in the network.
Clustering coefficient	The sum of the proportions of nodes that are directly connected to another node [38]. This parameter evaluates the average number of three nodes connected and measures how clustered the manure trade network is.

**Table 2:** Roles of respondents found in the manure trade network.

Role in the Manure Trade Network	Number (%)	Definition
Beef farm owner	154 (25.20%)	A person who raises animals on a beef farm
Dairy farm owner	407 (66.61%)	A person who raises animals on a dairy farm
Tradesman	36 (5.89%)	A person who buys manure from farmers and sells manure to the final purchaser
Final purchaser	14 (2.30%)	A person who buys and uses manure for his/her crops

**Table 3:** Frequency of trading, distance from tradesman origin to cattle farm and to final purchaser.

District	Frequency of Trading (Time/Month) and Distance from Tradesman Origin to Cattle Farm (Kilometers)				Frequency of Trading (Time/Month) and Distance from Tradesman Origin to Final Purchaser (Kilometers)			
	Mean ± sd	Mean ± sd	Min.	Max.	Mean ± sd	Mean ± sd	Min.	Max.
Mae On	0.5 ± 0.18	16.3 ± 23.4	0.02	131.3	0.48 ± 0.16	33.7 ± 46.3	0.09	160.4
San Kamphaeng	0.39 ± 0.13	4.37 ± 5.36	0.008	43.9	0.49 ± 0.15	30.2 ± 38.4	0.32	84.1
San Sai	0.44 ± 0.17	22.1 ± 28.4	0.35	93.1	0.43 ± 0.17	3.3	3.3	3.3

**Table 4:** Network parameters of the types of nodes in the manure trade network involved in this study.

Parameters	Beef Farm Owner (*n* = 154)	Dairy Farm Owner (*n* = 407)	Tradesman (*n* = 36)	Final Purchaser (*n* = 14)
Mean in-degree (range)	0	0.0025 (0–1)	13.6 (1–71)	10 (1–27)
Median in-degree (Q1,Q3)	0 (0,0)	0 (0,0)	5 (2,15.8)	7.5 (3.25,14.2)
Mean out-degree (range)	1	1.08 (1–2)	0.88 (0–4)	0
Median out-degree (Q1,Q3)	1 (1,1)	1 (1,1)	1 (0,1)	0 (0,0)
Mean In-degree (weighted, α = 0.5, range)	0	0.0015 (0–0.6)	6.63 (0.25–38.39)	4.64 (0.3–12.35)
Median In-degree (weighted, α = 0.5, Q1,Q3)	0 (0,0)	0 (0,0)	2.41 (1.03,8.12)	3.1 (1.8,6.93)
Mean Out-degree (weighted, α = 0.5, range)	0.32 (0.25–0.6)	0.58 (0.2–1.8)	0.46(0–1)	0
Median Out-degree (weighted, α = 0.5, range)	0.33 (0.25,0.33)	0.6 (0,0.6)	0.5 (0,1)	0 (0,0)
Mean betweenness (range)	0	0.0025 (0–1)	13.42 (0–114.5)	0
Median betweeness (Q1,Q3)	0 (0,0)	0 (0,0)	2 (0,6)	0 (0,0)
Mean closeness (×10^−6^) (range)	2.69 (2.69–2.7)	2.69	2.68	2.68
Median closeness (×10^−6^) (Q1,Q3)	2.69 (2.69,2.69)	2.69 (2.69,2.70)	2.69 (2.68,2.69)	2.68 (2.68,2.68)
Mean K-*core* (range)	1	1.1 (1–2)	1.58 (1–2)	1.5 (1–2)
Median K-*core* (Q1,Q3)	1 (1,1)	1 (1,2)	2 (1,2)	1.5 (1,2)

Q1 = first quartile, Q3 = third quartile

**Table 5:** Dyad census of a manure trade network in the three studied districts.

Type of Dyads	Number of Pairs
Mutual (A<->B)	3
Asymmetric (A->B)	623
Null	185,729

A, B = an individual node in the network

**Table 6:** Triad census of a manure trade network in the three studied districts.

Type of Triad	Number of Triads
A, B, C (empty graph)	37,460,585
A->B, C (graph with a single directed tie)	359,556
A<->B, C (graph with a mutual connection between two nodes)	0
A<-B->C (out-star)	39
A->B<-C (in-star)	9511
A->B->C (directed line)	371
A<->B<-C	0
A<->B->C	0
A->B<-C, A->C	3
A<-B<-C, A->C.	0
A<->B<->C.	0
A<-B->C, A<->C.	0
A->B<-C, A<->C.	0
A->B->C, A<->C.	0
A->B<->C, A<->C.	0
A<->B<->C, A<->C	0

A, B, C = an individual node in the network.

## Supplementary Materials

The following are available online at https://www.mdpi.com/2306-7381/7/3/138/s1, Questionnaire template and consent form were deposited at figshare; https://doi.org/10.6084/m9.figshare.12966641.v1.
