# Supplementary material for: Cattle Manure Trade Network Analysis and the Relevant Spatial Pathways in an Endemic Area of Foot and Mouth Disease in Northern Thailand
Source: Vet Sci. 2020 Sep 19;7(3):138. doi: 10.3390/vetsci7030138 (PMC7557812; doi:10.3390/vetsci7030138)
Supplement: Supplementary file 1 [file vetsci-07-00138-s001.zip › vetsci-910784-s/consent from.docx]

AF 03-09

Faculty of Veterinary Medicine, Chiang Mai University

Chiang Mai 50100, Thailand Phone+66 53948075

**Consent Form**

**Date of Consent** Date………………………….Month……………………………..Year

I am Miss/Mr./Mrs………………………………………………..age……………………………

Address……………………………………………………………………………………………..........

I read all information about this research “The Statistical Modeling of Foot and Mouth Disease Outbreak in Chiang Mai” and my participation is completely voluntary.

1. Before I sign this consent form, I was explained from the researcher about all objective and methodology and I clearly understand.
2. I answer all questions spontaneously and disclose any information to the researcher.
3. I am free to withdraw from the study at any time and my participation is completely voluntary.
4. The researcher will keep your personal information as secret. The researcher show only conclusion of the research.
5. I read all information above, understand all content and sign this consent from voluntary.

…………………………………… interviewee

…………………………………..

(…………………………………)

Date……month…..year………
