# Supplementary material for: Cattle Manure Trade Network Analysis and the Relevant Spatial Pathways in an Endemic Area of Foot and Mouth Disease in Northern Thailand
Source: Vet Sci. 2020 Sep 19;7(3):138. doi: 10.3390/vetsci7030138 (PMC7557812; doi:10.3390/vetsci7030138)
Supplement: Supplementary file 1 [file vetsci-07-00138-s001.zip › vetsci-910784-s/Template of questionnaire.docx]

**Questionnaire1: Questionnaire Sheet Used to Record Information during Dairy Farm Visits**

Date………………….............Subdistrict...................................................District……………………

X-Y coordinates .................. lat……………………… long………………………………

**Farmer information** Name…………………………………………………………………………age…………year

Gender ( ) Male ( ) Female Experience...........year Number of animal.....................heads

Education ( ) primary school ( ) secondary school ( ) undergraduate ( ) other…… Address…………………………………………………………………Tel.................................

Type of Farm ( ) beef farm ( ) dairy farm; Milk collecting center..................... ID member………

1. **Farm husbandry practices**
   1. Type of Farming

…………………………………………………………………

1.2 Distance between farm and neighboring farm

( ) dairy farm...................................................

( ) beef farm...................................................

1.3 Type of water source in your farm ( ) tap water ( ) other sources ................................

1.4 Farm located near roadways ( ) No ( ) Yes X-Y coordinates.....................................................

1.5 Dairy farm managed waste production (i.e., cow dung, contaminated runoff) to prevent waste from accumulating in feeding, watering, and shade areas

( ) No ( ) Yes.....................................................

1.6 Farms located within a 5 km radius of slaughterhouse

( ) No ( ) Cattle abattoir ( ) Pig abattoir ( ) X-Y coordinates..........................................

1.7 Farms located near shared cattle grazing areas in a 10 km radius

( ) No ( ) Yes X-Y coordinates.....................................................

1.8 Farms located within a5 km radius of milk collecting center

( ) No ( ) Yes X-Y coordinates.....................................................

**2. Animal and vehicle movement**

2.1. Purchasing of a new cow and/or young stock without following Quarantine protocol

( ) No ( ) Yes ...............................................days

Number of new animal ……heads/time Date of activity....................... Name..................................address..................................................tel..............................

Number of new animal ……heads/time Date of activity....................... Name..................................address..................................................tel................................

2.2. Entrance of vehicle carrying the young stock and/or cow out of the farm

Number of animal ……heads/time Date of activity....................... Name..................................address..................................................tel................................

Number of new animal ……heads/time Date of activity....................... Name..................................address..................................................tel................................

2.3. Entrance of vehicle carrying commercial feed for delivery

Frequency of activity ……time/week Date of activity....................... Name..................................address..................................................tel................................

Frequency of activity ……time/week Date of activity....................... Name..................................address..................................................tel................................

Frequency of activity ……time/week Date of activity....................... Name..................................address..................................................tel................................

2.4. Entrance of vehicle carrying roughage feed for delivery

Frequency of activity ……time/week Date of activity....................... Name..................................address..................................................tel................................

Frequency of activity ……time/week Date of activity....................... Name..................................address..................................................tel................................

2.5. Entrance of cattle manure trader vehicles

Frequency of activity ……time/week Number of bag…….bags/time Date of activity.................... Name..................................address..................................................tel................................

Frequency of activity ……time/week Number of bag…….bags/time Date of activity.................... Name..................................address..................................................tel................................

2.6. Using artificial insemination (AI) service

Frequency of activity ……time/week Date of activity....................... Name..................................address..................................................tel................................

Frequency of activity ……time/week Date of activity....................... Name..................................address..................................................tel................................

2.7. Type of raw milk transportation from farm to milk collecting center

( ) own farm vehicle ( ) publicly shared milk collecting vehicles Name..................................address..................................................tel................................ Name..................................address..................................................tel................................ Name..................................address..................................................tel................................ Name..................................address..................................................tel................................ Name..................................address..................................................tel................................

**3. Historical of FMD and vaccination status**

3.1 Vaccination was done within 4 months before FMD outbreak Date...........................

( ) All ( )No reason..........................................................................................

3.2 FMD vaccination administration

( ) Owner

( ) Animal health volunteer Name.....................................................................................

( ) DLD Name...........................................................................................................

( ) Dairy cooperative staff members Name ……………………………………………

3.3 Historical of FMD

1. FMD outbreak status in the previous 12 months

( ) No ( ) Yes

Time ...... ( ) Date............................sick animal.............heads herd.............heads

Time ...... ( ) Date............................sick animal.............heads herd.............heads

2. FMD outbreak in your neighboring in this area

( ) No ( ) Yes

Time ........ ( ) Date of outbreak..................................date of recovery..........................

Time ........ ( ) Date of outbreak..................................date of recovery..........................

**4. Farm biosecurity during outbreak**

4.1 Using disinfectant for vehicle and floor cleaning ( ) No ( ) Yes type...................................

4.2 Treatment of FMD infected cattle ( ) No ( ) Yes How.....................................................

4.3 FMD infected carcass management ( ) No ( ) Yes How.....................................................

4.4 Farm biosecurity when FMD outbreak ........................................................................................................................................

4.5 Chanel of communication .............................................................................................................................................

**Questionnaire2: Questionnaire Sheet Used to Record Information during Trader Visits**

Date………….............Subdistrict...................................................District……………………

XY point.................. lat……………………… long………………………………

Name…………………………………………………………………………age…………year

Gender ( ) Male ( ) Female Experience...........year Number of animal............................heads

Education ( ) primary school ( ) secondary school ( ) undergraduate ( ) other………...

Address………………………………………………………………………Tel...........................

**Trade information**

1. Type of trader

( ) Cattle Frequency of activity ………time/week

( ) Commercial feed Frequency of activity ………time/week

( ) Roughage feed Frequency of activity ………time/week

( ) Cattle manure Frequency of activity ………time/week

( ) AI Frequency of activity ………time/week

( ) Milk Frequency of activity ………time/week

2. Type of trading: buyer Name..................................address.....................................................tel................................ Name..................................address.....................................................tel................................ Name..................................address.....................................................tel................................ Name..................................address.....................................................tel................................ Name..................................address.....................................................tel................................ Name..................................address.....................................................tel................................ Name..................................address.....................................................tel................................ Name..................................address.....................................................tel................................ Name..................................address.....................................................tel................................ Name..................................address.....................................................tel................................

3. Type of trading: Seller Name..................................address.....................................................tel................................ Name..................................address.....................................................tel................................ Name..................................address.....................................................tel................................ Name..................................address.....................................................tel................................ Name..................................address.....................................................tel................................ Name..................................address.....................................................tel................................ Name..................................address.....................................................tel................................ Name..................................address.....................................................tel................................ Name..................................address.....................................................tel................................ Name..................................address.....................................................tel................................

4. Biosecurity information

4.1 Using disinfectant for vehicle and floor cleaning

( ) No ( ) Yes type.....................................................

5. Please draw your route
